# Effects of aquatic exercise on mood and anxiety symptoms: A systematic review and meta-analysis

**DOI:** 10.3389/fpsyt.2022.1051551

**Published:** 2022-11-17

**Authors:** Zhengyan Tang, Ye Wang, Jingmin Liu, Yujie Liu

**Affiliations:** ^1^Division of Sports Science and Physical Education, Tsinghua University, Beijing, China; ^2^Lang Ping Research Center for Sports Culture and Policy, Beijing Normal University, Beijing, China

**Keywords:** swimming, mental health, anxiety, depression, aquatic exercise, mood

## Abstract

**Objective:**

Exercise has beneficial effects on mood and anxiety symptoms. However, the impact of aquatic exercise on mood and anxiety symptoms has not been clearly confirmed. Therefore, this study aimed to synthesize and systematically analyze evidence available on boosting mental health through aquatic exercise.

**Method:**

A systematic review and meta-analysis were conducted under the PRISMA 2020 guidelines. PubMed, BIOSIS Previews, PsycINFO, Medline, SPORTDiscus, Education Source, and Web of Science Core Collection (WoSCC) were searched in May 2022. The research included the influence of aquatic exercises on mood and anxiety symptoms. After assessing trial quality and completing data extraction, a meta-analysis was carried out through R software. The results were presented as a standardized mean difference (SMD) and the corresponding 95% confidence interval.

**Results:**

A total of 18 original trials were included. People who received aquatic exercise intervention had a statistically significant reduction in mental disorder symptoms compared with before. The results were aquatic exercise [SMD = −0.77, 95% CI (−1.08, −0.47), I^2^ = 77%, *P* < 0.01], swimming [SMD = −0.51, 95% CI (−1.14, 0.12), I^2^ = 78%, *P* < 0.01], aquatic aerobics [SMD = −0.92, 95% CI (−1.32, −0.53), I^2^ = 78%, *P* < 0.01], moderate intensity [SMD = −0.75, 95% CI (−1.07, −0.43), I^2^ = 67%, *P* < 0.01], and low intensity [SMD = −1.07, 95% CI (−1.08, −0.47), I^2^ = 85%, *P* < 0.01].

**Conclusion:**

Aquatic exercise could statistically significantly improve mental health. Light aquatic aerobics probably has a better effect on mood and anxiety symptoms. However, given the number and quality of included research, verifying the aforementioned conclusions requires a larger sample of high-quality studies.

## Introduction

Mental health is critically important to everyone, everywhere (1). Mood and anxiety significantly diminish the quality of life and happiness of those who suffer from them (2). Some psychological problems become so severe that they can even lead to suicide. More than 90% of suicides in the West have been attributed to mood disorders (3). The prevalence of these conditions has a significant economic impact on society because of the difficulties they cause for affected individuals on a daily basis (4–6). One study from China found that in January and February 2020, 54% of subjects expressed psychological symptoms as severe or moderate, 29% of subjects reported moderate to serious anxiety, and approximately 17% of subjects reported moderate to severe depression (7). Therefore, finding some effective and acceptable intervention methods that can improve mood and anxiety symptoms is significant.

As a non-drug treatment for mental disorders, exercise has become the focus of more and more researchers' attention. Recent studies support that exercise, especially aerobic exercise (8) and physical activity, has beneficial effects on mental health (9). Exercise was promoted as the first level in the Canadian Clinical Guidelines 2016 emotional therapy (10).

Aquatic exercise, as a special aerobic exercise, has been shown to potentially benefit mood and anxiety (11–14). Aquatic exercise has many physiological benefits compared to land-based workouts because of the water's unique properties, such as buoyancy, pressure, resistance, and protection from skin irritation due to temperature and touch (15). As a result, when compared to other forms of exercise, aquatic exercise may prove to be the most effective in terms of its positive impact on mood. However, to our knowledge, no study has been conducted to synthesize the research on the psychological benefits of aquatic exercise. A systematic review of the studies and a meta-analysis are necessary to elaborate on the effect of aquatic exercise on mood and anxiety.

Furthermore, the effect of exercise on mental health is affected by many factors, including differences in individual characteristics and specific parameters of exercise intervention. Aging of both the body and mind may be associated with an increased probability of developing mood disorders (16). Exercise as an intervention to treat chronic diseases is associated with mood elevations in patients with various chronic diseases and disabilities (17). The type, intensity, and duration of exercise also affect the effect of exercise on mood. Specifically, long-time relaxing aerobic exercise may promote greater mood benefits (18–20). Although little evidence supports greater mood improvements in response to exercise among women, (21) some previous studies showed that exercise (dance, yoga, aerobic games, etc.) reduced depressive symptoms, with no moderating effect of sex (22–25). However, it is unclear whether there are differences in age, sex, disease, intensity, and duration in the impact of aquatic exercise on mood.

This study aimed to synthesize and systematically analyze the available evidence to determine the effect of aquatic exercise on mental health.

## Methods

The systematic review and meta-analysis followed the guidance of the PRISMA 2020 statement (26).

### Eligibility criteria

The eligibility and inclusion criteria of the article are as follows: 1) The study design must only include randomized controlled trials and quasi-experimental studies. 2) The article's full text must be available. 3) The article must be written in English. 4) It must only include peer-reviewed journal articles. 5) The subjects must be limited to humans. 6) Interventions must have included any type of aquatic exercise. 7) Mental health, mood, anxiety symptoms, depression, or related parameters in the study could be clearly extracted.

Aquatic exercise in the encyclopedia was defined as “an activity,” and the activity site must be in the water, such as a pool, lake, or ocean (27). Based on this, all types of exercise in water (such as swimming, aquatic exercise, and floating in water) were included in this study. Additionally, demographic restrictions were waived.

### Information sources and search strategy

PubMed, BIOSIS Previews, PsycINFO, Medline, SPORTDiscus, Education Source, and Web of Science Core Collection were searched on May 28, 2022, for studies using the following combination of terms: “mental health,” “depression,” “anxiety,” “mood,” “POMS,” “BDI,” “BAI” in combination with “swim,” “aquatic,” “water sport,” and “aquatic exercise.” Table 1 shows the search strategies used for database searches (e.g., PubMed).

**Table 1:** Search strategy in PubMed.

**Step**	**Search strategies**
#1	“swim” OR “aquatic” OR “water sport” [Mesh]
#2	“swim” OR “aquatic exercise” OR “water sport” [Text Word]
#3	#1 OR #2
#4	“mental health” OR “depression” OR “anxiety” OR “mood” [Mesh]
#5	“mental health” OR “depression” OR “anxiety” OR “mood” [Text Word]
#6	“POMS” OR “BDI” OR “BAI” [Text Word]
#7	#4 OR #5 OR #6
#8	#3 AND #7

### Study selection

All of the search results were imported into Endnote. The duplicate was searched by year, title, and author. After removing duplicates, two authors (ZYT and JML) independently screened the studies based on the title and the abstract. Only experimental articles defining the effect of aquatic exercise on human mood and anxiety symptoms were included. Following the initial screening, two authors searched the full text and further evaluated the research according to the eligibility criteria. We resolved differences through discussion with another author (YW). The selection process of the study was exhibited by a PRISMA 2020 flow diagram.

### Assessment of trial quality and data extraction

Trial quality was assessed from the selected full-text articles by two authors—the studies' risk of bias was in accordance with the Cochrane Handbook for Systematic Reviews of Interventions. At the same time, the data for the article were extracted. The information from the included articles (author, date of publication, country, and study design), the characteristics of subjects (sample size, age, sex, health condition, etc.), and intervention (types of exercises, duration, frequency) were extracted from the included articles. Furthermore, outcomes measured (mean, SD) and measurement tools used (type of questionnaire) were extracted.

### Synthesis and analysis

The meta-analysis was performed by R 4.2.1 software (“meta” package). The Chi-square test was performed to determine whether or not there were statistically significant differences between the research results. Multiple similar homogenous studies were considered if *P* ≥ 0.1, I^2^ < 50%, and meta-analysis using a fixed-effect model. If *P* < 0.1, I^2^ ≥ 50%, the random-effects model was used. Because of the different ranges and measurement methods of mental health in these studies, standardized mean difference (SMD) and a 95% confidence interval (CI) were used for continuous data.

### Subgroup analysis

Subgroup analyses were grouped based on the following factors: age, disease, mode of exercise, type of mental health, duration of exercise, and intensity.

## Results

### Search strategy results

**Figure 1:**
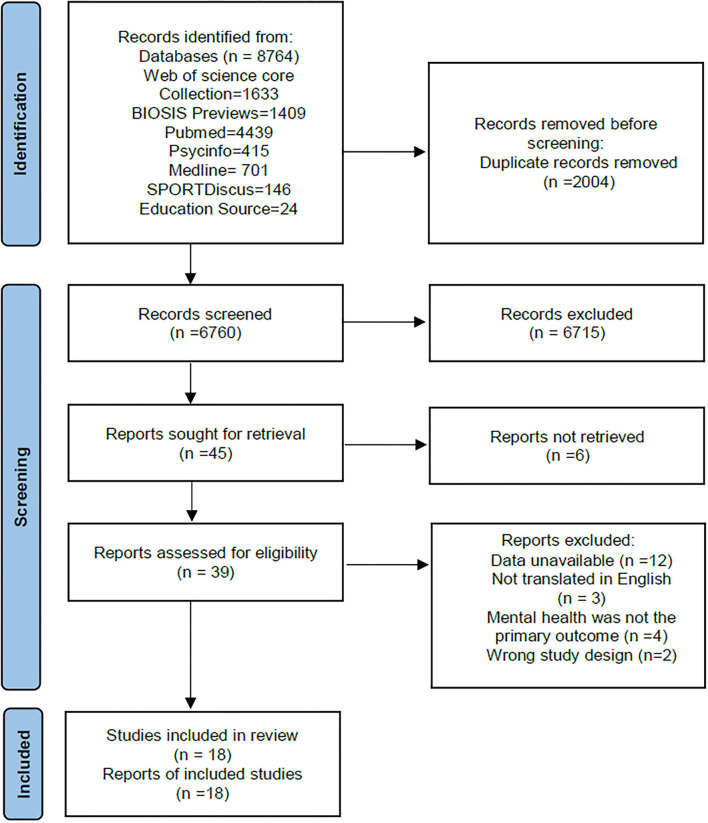
PRISMA flow chart. The figure shows the search strategy and selection of articles in this study (18 articles were included at the end).

The PRISMA flow chart (Figure 1) shows the search strategy and details the selection of articles for this review. A total of 8,764 articles were retrieved on a database search. After removing duplicates, 6,715 were removed through reviewing the title and abstract. Afterward, of the remaining 45 articles, through the full-text review, 27 were eliminated, and 18 were included.

### Study characteristics

Table 2 displays the general characteristics of the 18 articles, including the date of publication, the country, the study design, and the population of subjects.

**Table 2:** General characteristics.

**No**.	**Year**	**Author**	**Country**	**N**	**Population characters**	**Age**	**Mode**	**Measurement tool**	**Duration (week)**	**Frequency (d/week)**	**Intensity**	**Duration of sessions (min)**
1	1992	Berger and Owen. (28)	United States	39	College students	21	Swim	Profile of Mood State (POMS)	14	2	Moderate	40
2	1997	Berger et al. (29)	Australia	39	Swimmers	15	Swim	POMS	1	12	High	180
3	1999	Tanaka et al. (30)	United States	12	Obese subjects with stages 1 to 2 essential hypertension	48	Swim	POMS	10	3	Moderate	60
4	2001	Webb and Drummond. (31)	Australia	19	Beach swimming participants	26	Swim	Spielberger State-Trait Anxiety questionnaire	2	N/R^a^	Moderate	240
5	2002	Lindeman et al. (32)	Finland	25	Winter swimming	50	Winter swim	Crown Crisp Experimental Index (CCEI)	32	N/R	N/R	N/R
6	2004	Huttunen et al. (33)	Finland	36	Winter swimming	53	Winter swim	POMS	16	4	N/R	N/R
7	2015	Kim et al. (34)	Korea	25	Elderly women	72	Aquatic aerobics	POMS	24	3	Moderate	60
8	2016	Razazian et al. (35)	Switzerland		Female patients with multiple sclerosis	34	Aquatic aerobics	Beck's Depression Inventory (BDI)	8	3	Moderate	60
9	2018	Aidar et al. (36)	Brazil	19	Persons with stroke	52	Aquatic aerobics	StateTrait Anxiety Inventory & BDI	12	2	Moderate	60
10	2018	Da Silva et al. (37)	Brazil	29	Hypertensive adults & health adults	53	Aquatic aerobics	BDI & Beck's Anxiety Inventory (BAI)	12	2	light	45
11	2018	Delevatti et al. (38)	Brazil	17	Patients with type 2 diabetes	54	Aquatic aerobics	BDI	12	3	high	45
12	2019	da Silva et al. (39)	United States	30	Nondepression elderly & elderly with depression	58	Aquatic aerobics	BDI & BAI	12	2	light	45
13	2019	de Oliveira et al. (38)	Brazil	10	Elderly women	67	Swim	Geriatric Anxiety Inventory (GAI) & Perceived Stress Scale	12	2	light	45
14	2019	Perez et al. (40)	Brazil	10	Patients with Parkinson's disease	67	Aquatic aerobics	Short Geriatric Depression Scale	15	2	light	45
15	2019	Sahin et al. (14)	Turkey	30	People with osteoarthritis	63	Aquatic aerobics	Hospital Anxiety and Depression Scale (HAD)	3	5	Moderate	60
16	2020	Useros et al. (41)	Chile	15	People with cervical dystonia	48	Aquatic aerobics	BDI & State-Trait Anxiety Inventory (STAI)	4	1	light	50
17	2021	da Silva et al. (42)	United States	30	Health adults &people with Type 2 diabetes mellitus	64	Aquatic aerobics	BDI & BAI	12	0.5	light	41
18	2021	Lee et al. (43)	Korea	20	Pre-frailty elderly women	73	Aquatic aerobics	POMS	12	3	Moderate	60

a N/R, Not reported.

### Quality of the evidence

**Figure 2:**
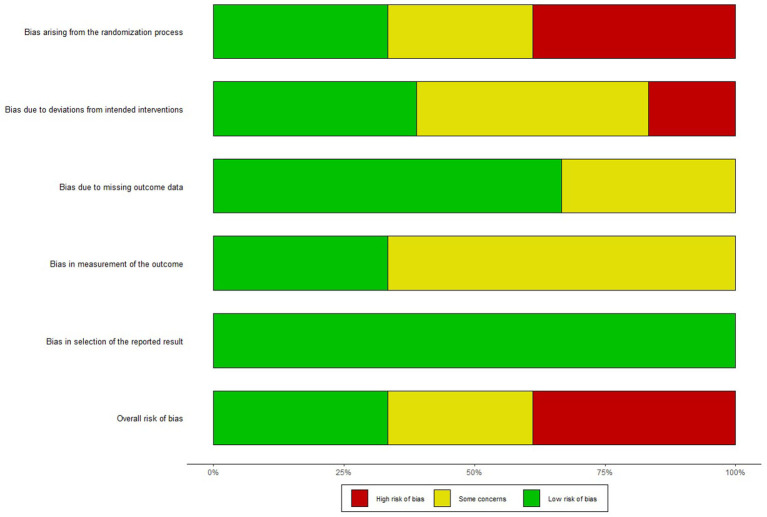
Risk of bias summary.

**Figure 3:**
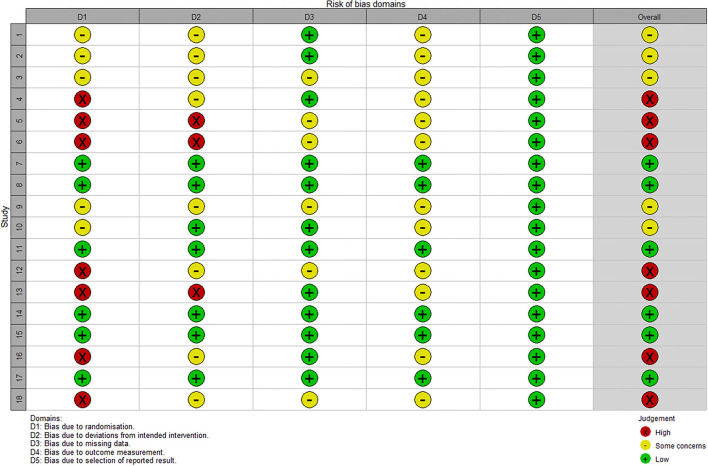
Risk of bias graph.

The assessment of trial quality was performed independently by two authors, according to Version 2 of the Cochrane risk-of-bias tool for randomized trials (RoB 2) (44). All discrepancies were discussed or consulted with another author. After evaluating the risk of bias in studies, seven studies were considered to have a high bias risk arising from the randomization process, three studies were found to have an ambiguous bias risk due to deviations from intended interventions, and 12 studies were found to have complete data (Figure 2). Overall, six studies have low risk, five have some concerns, and seven have a high risk (Figure 3).

### Effects of aquatic exercise on mental health

**Figure 4:**
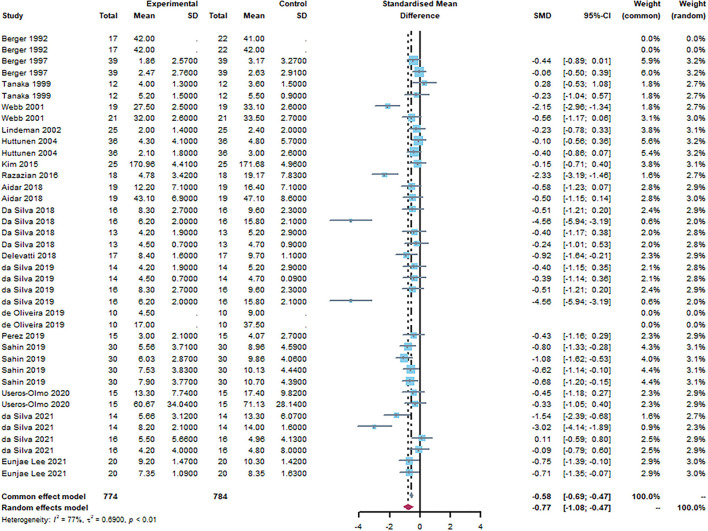
Forest plot of aquatic exercise effects on mood. The overall effect of aquatic exercise: [SMD = −0.77, 95% CI (−1.08, −0.47), I2 = 77%, *P* < 0.01].

According to meta-analysis, people who were treated with aquatic exercise showed a statistically significant reduction in mental disorder symptoms compared to pro-intervention [SMD = −0.77, 95% CI (−1.08, −0.47), I^2^ = 77%, *P* < 0.01]. As depicted in Figure 4, aquatic exercise improves mood and anxiety symptoms.

### Subgroup analysis

#### Subgroup: Age

**Figure 5:**
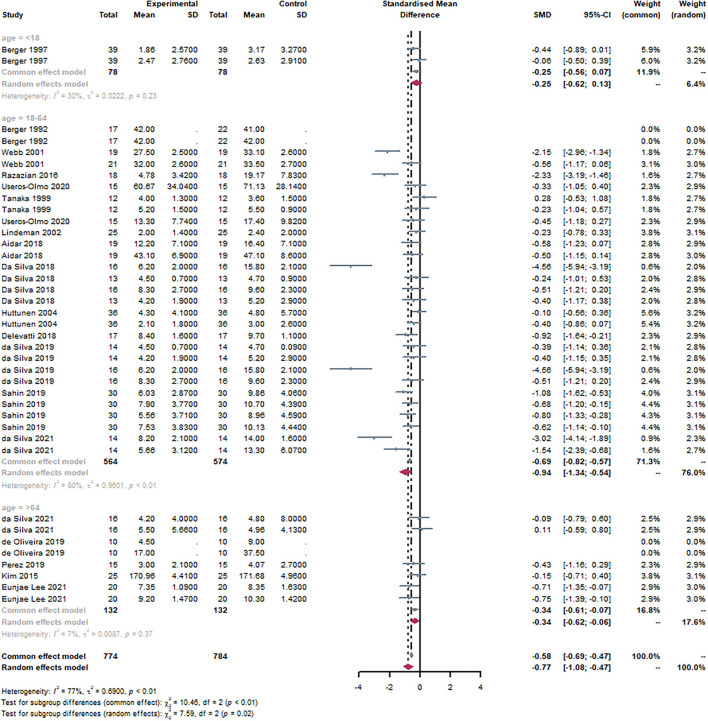
Forest plot of age. By subgroup analysis of the age, 18–64 years [SMD = −0.94, 95% CI (−1.34, −0.54), I^2^ = 80%, *P* < 0.01] were statistically significant.

Based on the age of the subjects, studies were divided into three groups, with an age range of <18 years, 18–64 years, and>64 years, respectively. Age groups were classified according to WHO standards (45). It can be observed that 18–64 years [SMD = −0.94, 95% CI (−1.34, −0.54), I^2^ = 80%, *P* < 0.01] were statistically significant through the subgroup analysis (Figure 5). There was no statistical significance in other groups. There was a statistically significant subgroup effect (*P* = 0.02).

#### Subgroup: Disease

**Figure 6:**
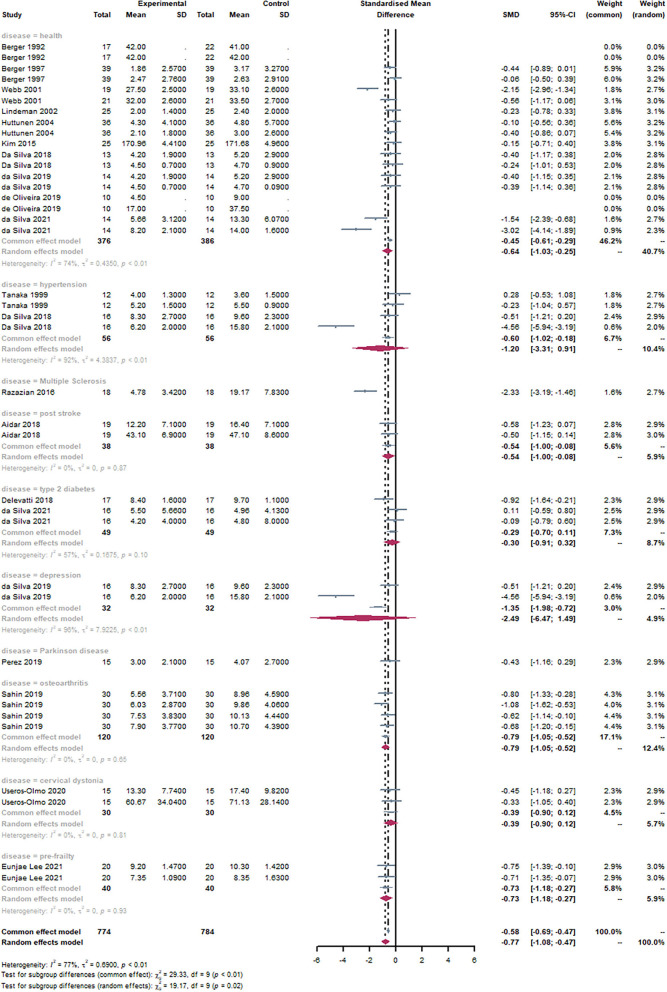
Forest plot of disease. By subgroup analysis of the disease, the Health group [SMD = −0.64, 95% CI (−1.03, −0.25), I^2^ = 74%, *P* < 0.01], Hypertension group [SMD = −1.20, 95% CI (−3.31, 0.91), I^2^ = 92%, *P* < 0.01], and Depression group [SMD = −2.49, 95% CI (−6.47, 1.49), I^2^ = 96%, *P* < 0.01] were statistically significant.

Studies were divided into ten groups based on the subjects' physical health. It can be observed that the Health group [SMD = −0.64, 95% CI (−1.03,−0.25), I^2^ = 74%, *P* < 0.01], Hypertension group [SMD = −1.20, 95% CI (−3.31, 0.91), I^2^ = 92%, *P* < 0.01], and Depression group [SMD = −2.49, 95% CI (−6.47, 1.49), I^2^ = 96%, *P* < 0.01] were statistically significant through the subgroup analysis (Figure 6). There was no statistical significance in other groups. There was a statistically significant subgroup effect (*P* = 0.02).

#### Subgroup: Mode of exercise

**Figure 7:**
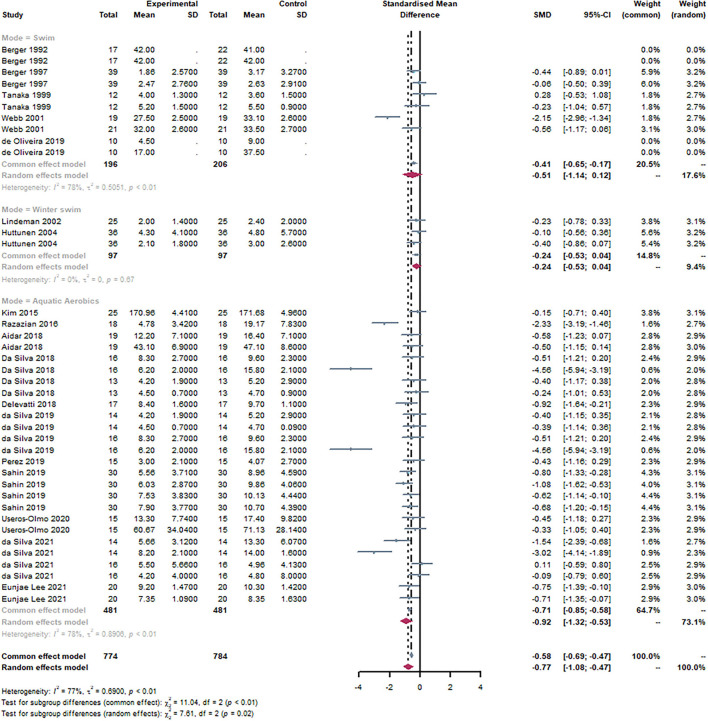
Forest plot of the mode of exercise. By subgroup analysis of the disease, swimming [SMD = −0.51, 95% CI (−1.14, 0.12), I^2^ = 78%, *P* < 0.01], and aquatic aerobics [SMD = −0.92, 95% CI (−1.32, −0.53), I^2^ = 78%, *P* < 0.01] were statistically significant.

Based on the exercise mode of intervention, studies were divided into three groups. The swimming group included swimming learning courses, leisure swimming, and swimming training. The aquatic aerobics group included water walking, water gymnastics, and any form of aerobic exercise in water, except swimming. It can be observed that swim [SMD = −0.51, 95% CI (−1.14, 0.12), I^2^ = 78%, *P* < 0.01] and aquatic aerobics [SMD = −0.92, 95% CI (−1.32, −0.53), I^2^ = 78%, *P* < 0.01] were statistically significant through the subgroup analysis (Figure 7). There was no statistical significance in the winter swim. There was a statistically significant subgroup effect (*P* = 0.02).

#### Subgroup: Type of mental health

**Figure 8:**
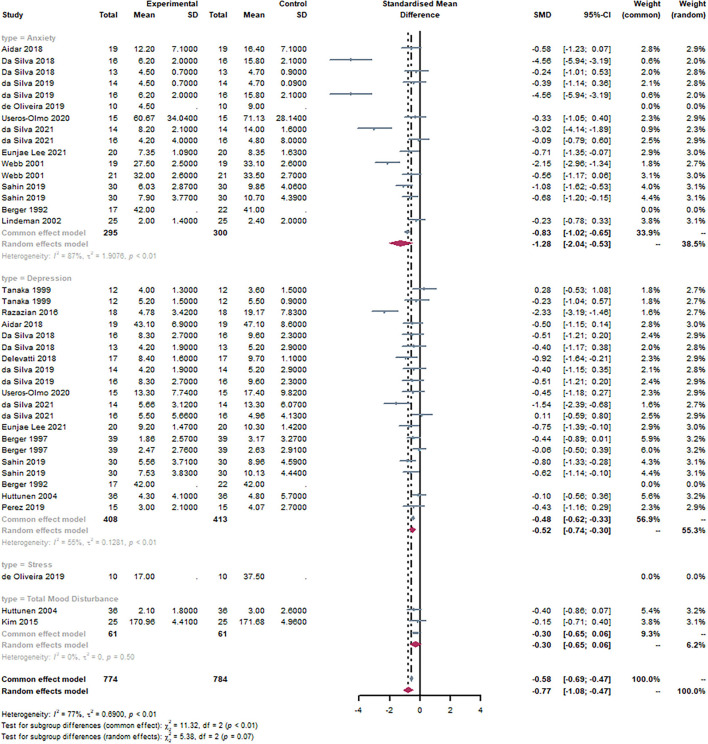
Forest plot of the type of mental health. By subgroup analysis of the disease, Anxiety [SMD = −1.28, 95% CI (−2.04, −0.53), I^2^ = 87%, *P* < 0.01] and Depression [SMD = −0.52, 95% CI (−0.74, −0.30), I^2^ = 55%, *P* < 0.01] were statistically significant.

Based on the type of mental health intervention, studies were divided into three groups. It can be observed that anxiety [SMD = −1.28, 95% CI (−2.04, −0.53), I^2^ = 87%, *P* < 0.01] and depression [SMD = −0.52, 95% CI (−0.74, −0.30), I^2^ = 55%, *P* < 0.01] were statistically significant through the subgroup analysis (Figure 8). There was a statistically significant subgroup effect (*P* = 0.07).

#### Subgroup: Duration

**Figure 9:**
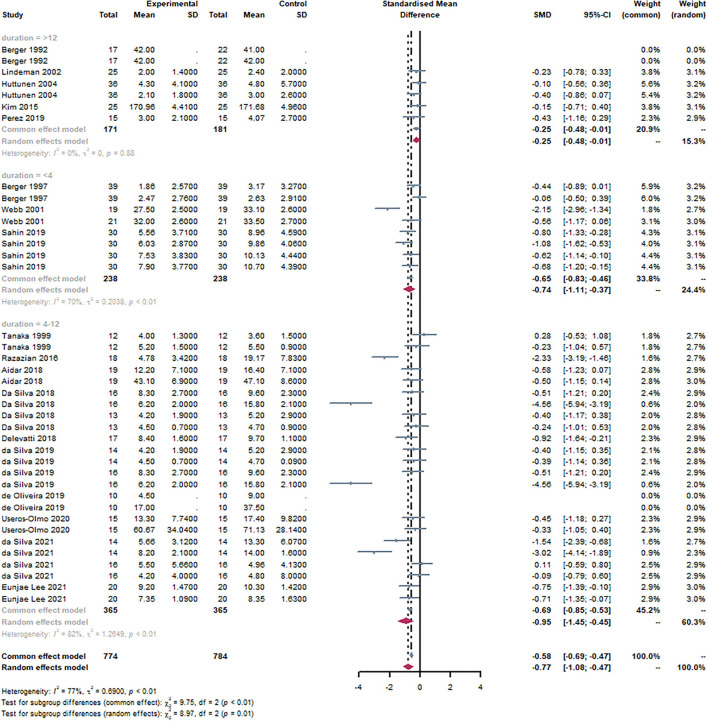
Forest plot of duration of exercise. By subgroup analysis of the disease, <4 weeks [SMD = −0.74, 95% CI (−1.11, −0.37), I^2^ = 70%, *P* < 0.01] and 4–12 weeks [SMD = −0.95, 95% CI (−1.45, −0.45), I^2^ = 82%, *P* < 0.01] were statistically significant through the subgroup analysis.

Based on the duration of the subjects, studies were divided into three groups, with an age range of <4 weeks, 4–12 weeks, and>12 weeks, respectively. It can be observed that <4 weeks [SMD = −0.74, 95% CI (−1.11, −0.37), I^2^ = 70%, *P* < 0.01] and 4–12 weeks [SMD = −0.95, 95% CI (−1.45, −0.45), I^2^ = 82%, *P* < 0.01] were statistically significant through the subgroup analysis (Figure 9). There was no statistical significance in the> 12-week group. There was a statistically significant subgroup effect (*P* = 0.01).

#### Subgroup: Intensity

Intensity was one of the most important parameters of exercise intervention. Heart rate, as the main indicator of intensity, was measured in most studies. Based on the report of the American College of Sports Medicine, < 64% of the maximum was considered low intensity, 64–76% of the heart rate maximum was regarded as moderate intensity, and 77–95% of the heart rate maximum was considered high intensity (46). However, a few studies did not report the measurement of intensity.

**Figure 10:**
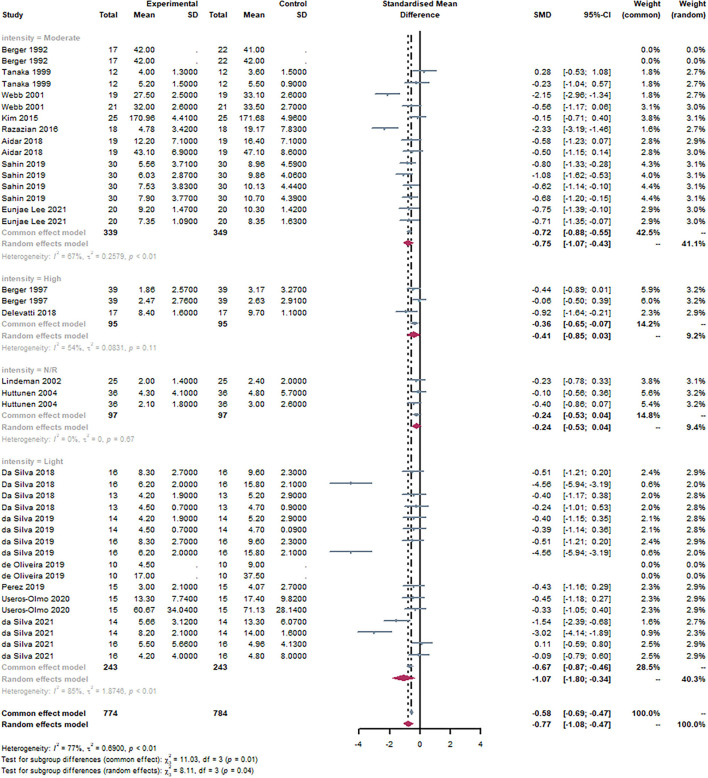
Forest plot of the intensity of exercise. By subgroup analysis of the disease, moderate [SMD = −0.75, 95% CI (−1.07,−0.43), I^2^ = 67%, P < 0.01] and light [SMD = −1.07, 95% CI (−1.08, −0.47), I^2^ = 85%, P < 0.01] were statistically significant through the subgroup analysis.

According to the intensity of the subjects, studies were divided into three groups, light, moderate, and high, respectively. It can be observed that moderate [SMD = −0.75, 95% CI (−1.07, −0.43), I^2^ = 67%, *P* < 0.01] and light [SMD = −1.07, 95% CI (−1.08, −0.47), I^2^ = 85%, *P* < 0.01] were statistically significant through the subgroup analysis (Figure 10). There was no statistical significance in the high group. There was a statistically significant subgroup effect (*P* = 0.04).

### Publication bias

**Figure 11:**
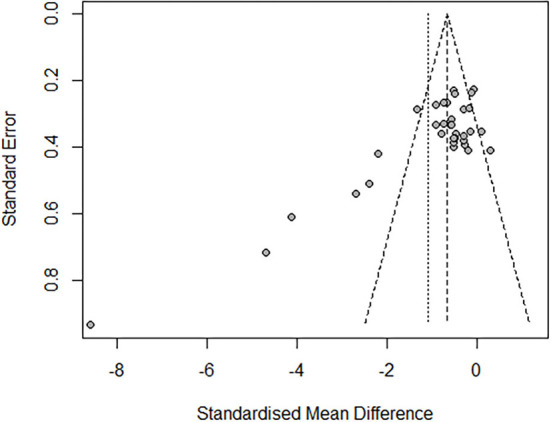
Funnel plot of publishing bias.

The funnel plot indicates the possible publication bias (Figure 11). Furthermore, the Egger method was used for analysis. When the linear regression test was *P* < 0.01, the publication bias of studies was statistically significant.

**Figure 12:**
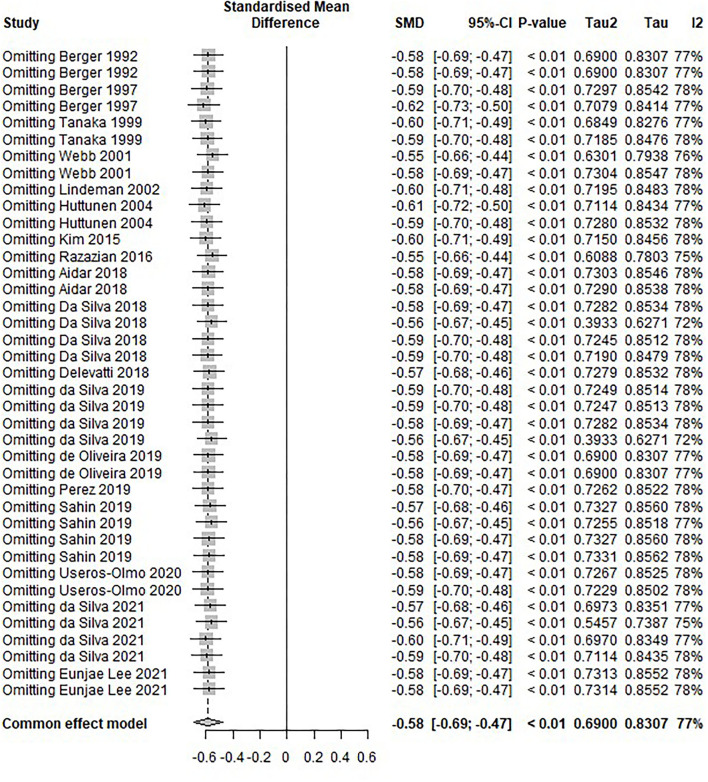
Sensitivity analysis plot. A leave-one-out meta-analysis was used to test the publication bias of a single study. No studies affecting heterogeneity were found (I^2^ = 72–78%).

A sensitivity analysis was also conducted. A leave-one-out meta-analysis was used to test the publication bias of a single study (Figure 12). After sequentially removing each study, no studies affecting heterogeneity were found (I^2^ = 72%−78%). This analysis confirmed the stability of the results.

## Discussion

This meta-analysis included 423 people who received aquatic exercise intervention; 18 studies synthesized the benefits of aquatic exercise, and the results revealed that aquatic exercise could statistically significantly improve mood and anxiety symptoms. The overall SMD = −0.77, [95% CI (−1.08, −0.47), I^2^ = 77%, *P* < 0.01].

Ten different states of physical health are included in this review. Few comparable studies of a particular disorder made it difficult to determine which states would reap the most benefits from aquatic exercise. Nevertheless, this review found that aquatic exercise may be effective for general states of physical health observed, especially depression and hypertension. It is possible that the exercise increases the secretion of the related release of b-endorphin and dopamine and provides a soothing effect (47), while the muscular resistance of the water is more than ten times higher than the resistance of land. Hence, it requires more activation of the motor cortex in the elderly (39). Besides, people with type 2 diabetes are usually not sufficiently active. Activation of the brain and mood improvement are potentially important motivators for exercise (48). For patients with hypertension, the levels of the cytokines (TNF-α and IL-6) could be reduced by participating in the aquatic exercise program, suggesting the inhibitory effect of aquatic exercise on the production of pro-inflammatory cytokines (37).

Aquatic exercise is particularly effective in reducing anxiety. Comparing the results of this study with other studies, a meta-analysis conducted by Song (49) found the effect of land-based aerobic exercise on anxiety (SMD:−0.50), traditional Chinese exercise (SMD:−0.03), and meditation (SMD: −0.15). The above three exercises are lower than aquatic exercises (SMD: −1.28). Several studies have also shown that aquatic exercise can boost mood (35, 40, 50). However, those studies were relatively narrow, focusing primarily on relatively menial land aerobic exercise. The effect of aquatic exercise is different from that of land exercise and needs further research.

Compared with land-based aerobic exercise, aquatic exercise shows its particularity. The sensation of water flowing through the skin when moving in water is difficult to obtain when moving on land. Several studies indicated that touch could reduce stress and improve mood (51–54). The reduction in gravity also reduces the load on the spine, knees, and other pain-prone areas. In addition to physical factors, aquatic exercises are difficult for some people, especially in the sea and other special environments; thus, “confronting challenges” was key to the impact of mood (55). Moreover, aquatic exercise serves to connect and convey a sense of nature. With the function of re-orientating and changing the sense of body, space, and gravity, people can expand their perspectives (56).

Regarding the intervention type of exercise, aquatic aerobics (SMD: −0.92) is better than swimming (SMD: −0.51), but these studies lack consistency. Swimming includes swim-learning programs (28, 38), swimming training for competition (29, 57), and leisure swimming (31, 58, 59). Swimming in different situations affects people's moods differently. Similarly, aquatic aerobics includes various forms. This may be one of the reasons for the high heterogeneity of research. In general, most of the aquatic aerobics' subjects are older or have ordinary diseases. Regarding safety and feasibility, older individuals prefer light, easy, and fun exercises over hard and stressful exercises (60). Therefore, they are more likely to benefit from water aerobics for their mental health than young, healthy adults. However, the trials in this study are insufficient for comparison. The specific impact needs further comparative study through similar samples. Winter swimming has little effect on improving mental health, which may be because the temperature stimulus of cold water is too strong, and then the body tends to remain tense (32, 33). For this reason, winter swimming may not be an effective way to improve mental health.

Based on the results of this study, the low intensity of aquatic exercise causes greater benefits for mental health. It may be that lower intensity makes people more relaxed (49). However, the acceptability of people of different ages and disease conditions to the intensity should also be considered, making it necessary to judge the research results carefully.

The influence of age and sex is not fully reflected in this study. In this study, the impact of exercise on the moods of the elderly was not statistically significant. The influence of age on the effect of exercise intervention is still controversial. A study of land-based exercise intervention shows that there were no significant differences in the amount of improvement between the younger and older exercise groups (61). However, some researchers find that exercise has greater distinct effects on brain activity and mood improvement in young people than in older people (62). Although the trials included in this investigation indicated the sex of the subjects, most studies were not classified by sex in the results, making sex-specific subgroup analysis challenging. Although some research (19, 25, 63, 64) suggests no difference in the positive effects of exercise based on sex, some evidence suggests that males may get greater advantages from exercise on their mood than females (65). Thus, more study is required to determine whether aerobic exercise has different effects on mood depending on gender and age.

There are a few limitations to this study. First, the between-study heterogeneity is significant, and our subgroup and sensitivity analyses cannot entirely account for it. Second, this study only selected articles published in English to limit the risk of bias. There may be influential publications written in languages other than English that were not included. Finally, non-randomized controlled trials were included, which may lead to selection bias. Given the limitations in our review, more large-scale research should be conducted in the future.

## Conclusion

Aquatic exercise could statistically significantly improve mental health. Light aquatic aerobics may have a better effect on mood and anxiety symptoms. However, given the number and quality of included research, verifying the above conclusions requires a larger sample size of high-quality studies.

## Data availability statement

The original contributions presented in the study are included in the article/supplementary material, further inquiries can be directed to the corresponding author.

## Author contributions

ZT and YW conceived of the idea and designed the study protocol. YL conducted statistical analysis. ZT and JL drafted the manuscript. ZT, YW, JL, and YL participated in the revision of the manuscript of the study. All authors contributed to the article and approved the submitted version.

## Funding

This study was funded by the Tsinghua University Initiative Scientific Research Program 2021THZWJC15.

## Conflict of interest

The authors declare that the research was conducted in the absence of any commercial or financial relationships that could be construed as a potential conflict of interest.

## Publisher's note

All claims expressed in this article are solely those of the authors and do not necessarily represent those of their affiliated organizations, or those of the publisher, the editors and the reviewers. Any product that may be evaluated in this article, or claim that may be made by its manufacturer, is not guaranteed or endorsed by the publisher.
